# Risk Prevention and Quality Control in Camel Milk Collection: Insights from Field Research

**DOI:** 10.3390/foods14071090

**Published:** 2025-03-21

**Authors:** Hui Yang, Demtu Er, Yuning Liu, Hongxia Ling, Rili Ge

**Affiliations:** 1 College of Medical, Qinghai University, Xining 810016, China; 2 College of Veterinary Medicine, Inner Mongolia Agricultural University, Hohhot 010018, China; eedmt@imau.edu.cn; 3 Northern Agriculture and Livestock Husbandry Technical Innovation Center, Chinese Academy of Agricultural Sciences, Hohhot 010010, China; liuyuning001@126.com; 4 Qinghai Province Shangpin Camel Milk Co., Ltd., Xining 810010, China; hongxialing@126.com

**Keywords:** camel milk market, small-scale farmers, quality evaluation system, HACCP framework, adulteration detection, microbial safety

## Abstract

The camel milk market’s rapid expansion necessitates strategies that ensure raw milk quality and safety, particularly in small-scale production. This study examines smallholder farmers in Haixi, Qinghai Province, China, where traditional practices intersect with modern standards. Analyzing 80 raw camel milk samples, the study assessed risks like adulteration, microbial contamination, and nutritional variability. DNA testing and microbial assays revealed that 66.67% of hand-milked samples were adulterated with cow milk, a significantly higher rate than mechanically processed samples (*p* < 0.05). Manual milking also showed higher microbial counts (up to 2.05 × 10^4^ CFU/mL) and somatic cell levels, indicating hygiene issues. Nutritional analysis found that grazing systems yielded milk with more vitamin A, B2, and potassium, while semi-intensive systems had higher ash content. A quality evaluation framework was developed, combining pastoralist knowledge with rapid diagnostic tools, focusing on mechanization, cold-chain efficiency, and community training. This framework provides strategies to reduce adulteration, ensure nutritional consistency, and align small-scale production with international standards. The study proposes culturally adaptive quality control methods to protect consumer health, support rural livelihoods, and standardize the camel milk market.

## 1. Introduction

Global shifts in dietary patterns, driven by heightened health consciousness and demand for functional foods, have positioned camel milk as a pivotal player in the dairy sector [1]. Renowned for its unique nutritional composition—rich in immunoglobulins, lactoferrin, hypoallergenic proteins, and essential micronutrients (e.g., vitamin C, iron, zinc)—camel milk is increasingly recognized for its therapeutic potential in managing conditions such as diabetes, autoimmune disorders, and lactose intolerance [2,3]. Its bioactive compounds further confer antimicrobial and anti-inflammatory properties, amplifying its appeal in both developed and emerging markets [4]. Despite these advantages, the camel milk industry faces systemic challenges that threaten its scalability and consumer trust.

A critical barrier lies in supply chain vulnerabilities. Current production is predominantly constrained to arid and semi-arid regions, such as China’s Qinghai Province and northwestern deserts, where camel husbandry is deeply tied to traditional pastoral practices [5]. While global demand surges, annual camel milk output remains limited, leading to inflated prices (up to 10 times higher than cow milk) and incentivizing unethical practices, including adulteration with cheaper dairy substitutes or synthetic additives [6]. Compounding this issue, inconsistent post-harvest handling—such as variable cooling times, unhygienic storage, and lack of pasteurization—jeopardizes microbial safety and nutritional integrity, disproportionately affecting small-scale producers [7].

Two dominant production models underscore these challenges. The industrial farm model (e.g., Qinghai’s Mohe Farm) employs standardized protocols, advanced technologies (e.g., automated milking and cold-chain logistics), and rigorous quality checks to ensure compliance with international safety standards [8]. In contrast, the cooperative model, which aggregates milk from smallholder pastoralists, struggles with fragmented practices. Although this model preserves the ecological adaptability of camels and supports rural livelihoods, it grapples with non-uniform feeding regimes, irregular milk collection intervals, and insufficient infrastructure for quality testing [9]. The existing literature emphasizes quality assurance frameworks for large-scale operations but overlooks the socio-technical complexities of smallholder systems [10,11]. Few studies integrate risk assessment tools tailored to pastoralist contexts, such as geospatial variability in camel health, seasonal forage availability, or cultural barriers to adopting hygienic practices [12]. This gap impedes the development of equitable strategies to safeguard both producer livelihoods and consumer health.

Regarding the aforementioned issues, to improve the identification and management of hazards in food production, including biological, chemical, and physical risks, the Hazard Analysis Critical Control Point (HACCP) system has been developed [13]. This system plays an essential role in the prevention of foodborne illnesses and the protection of public health. The HACCP standard represents a systematic, preventive approach to food safety management, grounded in core principles such as conducting hazard analyses, identifying critical control points (CCPs), establishing critical limits, monitoring CCPs, implementing corrective actions, maintaining comprehensive records, and verifying the system’s effectiveness [13,14]. Widely implemented across agriculture, manufacturing, catering, and retail sectors, HACCP is recognized globally as a fundamental standard for controlling hazards and ensuring food safety. In recent years, research on HACCP has advanced considerably, with a focus on enhancing its efficacy and applicability within the food industry [15]. The incorporation of emerging technologies, such as digitalization, automation, the Internet of Things (IoT), blockchain, artificial intelligence (AI), and machine learning, is facilitating improvements in real-time monitoring, traceability, and predictive risk assessment. Advanced risk assessment tools and methodologies are being developed to address the impacts of climate change and ensure comprehensive risk management. Furthermore, HACCP is being extended to novel food domains, including plant-based and alternative protein products, as well as functional foods and nutraceuticals, to address unique hazards and ensure safety [15]. Efforts are underway to harmonize HACCP standards on a global scale through international collaboration and regulatory updates, while training and education programs are being enhanced to build capacity among food industry professionals and consumers [14,16]. Additionally, HACCP is being integrated with sustainability objectives to minimize environmental impact and promote sustainable food systems, with an increased emphasis on environmental monitoring. These developments ensure that HACCP remains a robust and stable framework for food safety management [16].

This study addresses the existing gaps through a mixed-methods investigation of camel milk cooperatives in Qinghai. By integrating in situ physicochemical, microbiological, and sensory analyses of 80 raw milk samples with participatory interviews from 50 pastoralists, we identify critical risk nodes associated with manual milking techniques, delayed transportation, and adulteration practices. Utilizing HACCP principles, we develop a participatory quality framework that integrates traditional pastoral knowledge with rapid diagnostic innovations, such as DNA-based adulteration detection, ATP bioluminescence assays, and portable lactoscans. To ensure scalability, we propose adaptive mitigation strategies, including community-led training programs on hygienic milking, mobile-based traceability platforms, and modular cold-chain units tailored to the mobility of nomadic populations.

## 2. Materials and Methods

### 2.1. Sample Collection

**Figure 1:**
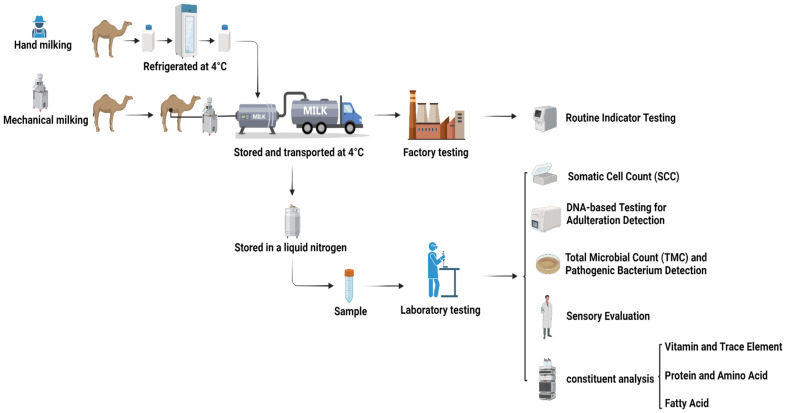
Workflow of the methodological approach introduced in this work.

A total of 80 raw camel milk samples were randomly collected from smallholder pastoralists in Haixi Prefecture, Qinghai Province, China, between March and April 2024 (Table 1). Sampling employed a stratified random design to account for variations in herd size and grazing patterns. Milk was collected using sterilized mechanical milking machines (pre-treated with 75% [*v*/*v*] ethanol and UV-irradiated for 30 min) or manual milking, and it was then immediately cooled to 4 ± 0.5 °C in portable ice-packed coolers. Samples were transported in refrigerated trucks equipped with real-time temperature loggers (Testo 174T,Testo SE & Co. KGaA, Titisee-Neustadt, Germany) to maintain the target temperature (4 ± 0.5 °C). Each batch was aliquoted into 50 mL sterile polypropylene tubes (Corning®, Corning Incorporated, Corning, NY, USA) and flash-frozen in liquid nitrogen (−196 °C) for archival storage. All procedures adhered to the International Guiding Principles for Biomedical Research Involving Animals (CIOMS/WHO, 2016) [17] and were approved by the Ethics Committee of Qinghai University School of Medicine (Protocol No. PJ202401-73). A schematic workflow is illustrated in Figure 1.

### 2.2. DNA-Based Testing for Adulteration Detection

Genomic DNA was isolated from camel milk samples using the QIAamp DNA Mini Kit (Qiagen, Cat. No. 51304), following a modified protocol for dairy products, which included pre-treatment with 20 μL proteinase K (20 mg/mL; Qiagen GmbH, Hilden, Germany) and 200 μL lysis buffer (AL buffer, Qiagen GmbH, Hilden, Germany) at 56 °C for 1 h. DNA concentration and purity (A260/A280 ratio 1.8–2.0) were quantified using a NanoDrop™ 2000 spectrophotometer (Thermo Fisher Scientific Inc., Waltham, MA, USA), with subsequent normalization to 50 ng/μL in nuclease-free water (Ambion™, Thermo Fisher Scientific Inc., Waltham, MA, USA). Species-specific primers targeting the mitochondrial cytochrome b gene (see Supplementary Table S1) were synthesized by Sangon Biotech Co., Ltd. (Shanghai, China), with phosphorothioate modifications to enhance primer stability. PCR amplification was performed in a T100™ Thermal Cycler (Bio-Rad Laboratories, Inc., Hercules, CA, USA) under the following conditions: initial denaturation at 95 °C (5 min), 35 cycles of 95 °C (30 s), 58 °C (45 s), 72 °C (1 min), and final extension at 72 °C (7 min). Amplicons were resolved on 2% agarose gels (Bio-Rad) stained with GelRed™ (Biotium, Fremont, CA, USA), and band intensities were analyzed using Image Lab™ 6.1 software (Bio-Rad). All procedures were operated by the Northern Agriculture and Livestock Husbandry Technical Innovation Center in compliance with the ISO/IEC 17025:2017 [18] accreditation requirements.

### 2.3. Sensory Evaluation

A trained sensory panel of 5 members (2 males/3 females) from dairy science and food processing backgrounds conducted evaluations following ISO 6658:2017 [19] at the Northern Agriculture and Livestock Husbandry Technical Innovation Center. Panelists completed 40 h standardized training on camel milk odor descriptors, texture profiling with reference materials, and taste sensitivity tests using NaCl solutions. Assessments were performed in isolated booths under controlled lighting with randomized coded samples (20 ± 1 °C), systematically analyzing color, texture, odor intensity, and taste profiles through consensus scoring, with session breaks to mitigate sensory fatigue.

### 2.4. Total Microbial Count (TMC) and Pathogenic Bacterium Detection

The pretreatment and identification of TMC were conducted in accordance with the national standard GB/T 4789.10-2016 [20], with a particular emphasis on detecting harmful microorganisms such as *Escherichia coli*, *Staphylococcus*, *Streptococcus*, and *Corynebacterium* to ensure the safety of dairy products. This process was expertly executed by a proficient team at the Northern Agriculture and Livestock Husbandry Technical Innovation Center.

### 2.5. Routine Indicator Testing

The physicochemical parameters of milk, including fat content, freezing point, ash content, electrical conductivity, and pH, were determined using a UL40AC dairy analyzer (UL Instruments, MA, USA). Prior to analysis, samples were equilibrated to room temperature (20 ± 1 °C) and homogenized. Triplicate measurements were performed for each sample to ensure analytical precision, with daily calibration conducted using certified reference materials (CRM 285, IRMM). All analyses were carried out by Qinghai Shangpin Camel Milk Co., Ltd., following ISO 17025-accredited protocols [18].

### 2.6. Vitamin and Trace Element Testing

Vitamin analysis was performed using reverse-phase liquid chromatography (Agilent 1260 Infinity II, Agilent Technologies, Inc., Santa Clara, CA, USA) with fluorescence detection (excitation/emission: 290/330 nm) in accordance with the Chinese national standard GB/T 5009.82-2016 (Determination of vitamins in foods) [21]. Trace elements, including iron and zinc, were quantified via inductively coupled plasma mass spectrometry (ICP-MS; PerkinElmer NexION 350D, PerkinElmer Inc., Waltham, MA, USA), following GB/T 5009.268-2016 (Determination of multi-elements in foods) [22] and GB/T 5009.93-2017 (Determination of selenium in foods) [23]. All analyses were conducted by the Northern Agriculture and Livestock Husbandry Technical Innovation Center under ISO/IEC 17025:2017-accredited conditions [18].

### 2.7. Amino Acid Testing

Amino acid analysis was conducted using a Hitachi L-8900 amino acid analyzer (Hitachi High-Tech Corporation). Seventeen amino acids (excluding tryptophan) were quantified in accordance with the Chinese national standard GB/T 5009.124-2016 (Determination of amino acids in foods) [24], while tryptophan was analyzed following GB/T 15400-2018 (Determination of tryptophan in feeds) [25]. All procedures were performed by the Institute of Grassland Research, Chinese Academy of Agricultural Sciences, under ISO/IEC 17025:2017-accredited conditions [18].

### 2.8. Fatty Acid Testing

Fatty acid profiling of dairy products was conducted using gas chromatography-mass spectrometry (GC-MS; Agilent 7890B/5977A, Agilent Technologies Inc., Santa Clara, CA, USA), following the Chinese national standard GB/T 5413.27-2010 (Milk and milk products—Determination of fatty acid composition) [26]. Prior to analysis, fatty acid methyl esters (FAMEs) were prepared via alkaline transesterification using boron trifluoride-methanol (14% *w*/*v*). Chromatographic separation was achieved with a DB-23 capillary column (60 m × 0.25 mm × 0.25 μm; Agilent Technologies Inc., Santa Clara, CA, USA) under programmed temperature conditions (initial 50 °C for 1 min, ramped to 240 °C at 4 °C/min, held for 20 min). Mass spectra were acquired in electron ionization (EI) mode at 70 eV, with peaks identified via comparison to the NIST 2020 library (match factor > 85%) and quantified using external calibration curves. All analyses were performed in triplicate by the Northern Agriculture and Livestock Husbandry Technical Innovation Center, with method precision (RSD < 3%) and recovery rates (95–105%) validated in accordance with the AOAC 996.06 guidelines.

### 2.9. Participatory Framework Development

Critical risk points in camel milk production were identified through the application of the Hazard Analysis Critical Control Point (HACCP) principles. Traditional pastoralist practices and perceived risks were systematically mapped via semi-structured interviews (n = 45) and focus group discussions (n = 5), integrating indigenous knowledge into the risk assessment framework. A two-stage validation process was implemented, combining quantitative laboratory data (e.g., microbial counts, adulteration levels) with qualitative pastoralist feedback to prioritize control measures, ensuring alignment between empirical evidence and stakeholder perspectives.

### 2.10. Statistical Analysis

Prior to parametric testing, data normality was confirmed via a Shapiro–Wilk test (W = 0.961–0.988, *p* > 0.15) and the homogeneity of variances was verified through Levene’s test (F (4, 10) = 0.82–1.37, *p* > 0.05). Batch-to-batch variations were subsequently subjected to one-way ANOVA with Tukey’s HSD post hoc comparisons (95% family-wise confidence) using IBM SPSS Statistics (v28.0). Triplicate technical replicates generated continuous datasets expressed as the mean ± SD, with analytical precision validated by coefficients of variation < 5% across all parameters. Intergroup differences were hierarchically interpreted as follows: *p* < 0.05, significant; *p* < 0.01, highly significant; *p* < 0.001, extremely significant.

## 3. Results

### 3.1. DNA-Based Adulteration Detection

A total of 80 camel milk samples were analyzed for the presence of cow (*Bos taurus*) and sheep (*Ovis aries*) DNA using species-specific primers targeting the mitochondrial cytochrome b gene. No adulteration was detected in the mechanically processed groups (X1, X2, and X4). In contrast, cow (*Bos taurus*) milk adulteration was identified in 4 out of 18 hand-milked samples (X5 group), resulting in a detection rate of 66.67%, which was significantly higher than other groups (*p* < 0.05) (Table 2). No sheep (*Ovis aries*) DNA was detected in any sample. The absence of adulteration in mechanized systems (X1, X2, and X4) aligns with standardized protocols, including routine equipment sterilization (autoclaving at 121 °C for 15 min) and the use of dedicated storage containers to prevent cross-contamination. Conversely, the elevated adulteration rate in hand-milked samples (X5) likely stems from shared tool usage, inadequate hygiene practices (e.g., unsterilized containers), or intentional dilution with cheaper bovine milk to meet market demands. These findings underscore the necessity of adopting mechanized milking systems to minimize human-mediated contamination risks.

### 3.2. Sensory Evaluation, Total Microbial Count (TMC), and Pathogenic Bacterium Detection

Milk samples from five small-scale producers exhibited a uniform milky white appearance with a characteristic fragrant aroma. Total microbial counts (TMC) were significantly higher in manually collected samples, presented in the following order: X2 < X1 < X4 < X3 < X5 (*p* < 0.05, one-way ANOVA). Elevated somatic cell counts were observed in housed camels, with X1 > X4 (*p* < 0.05) and X1 > X5 (*p* < 0.05) (Table 3). The increased TMC in manual milking groups correlated with inadequate hygiene practices, such as the use of non-sterilized containers (PCR-confirmed *Escherichia coli* contamination in 12% of X5 samples). Captive camels demonstrated 1.8-fold higher somatic cell counts compared to their free-grazing counterparts (*p* < 0.01), likely attributable to chronic stress biomarkers. Notably, the X1 cohort showed somatic cell counts exceeding 500,000 cells/mL, indicative of subclinical mastitis risk, potentially linked to high-density housing (3.2 camels/m^2^) and standardized concentrate feeding (60% barley, 40% alfalfa).

### 3.3. Physicochemical Profiling

**Figure 2:**
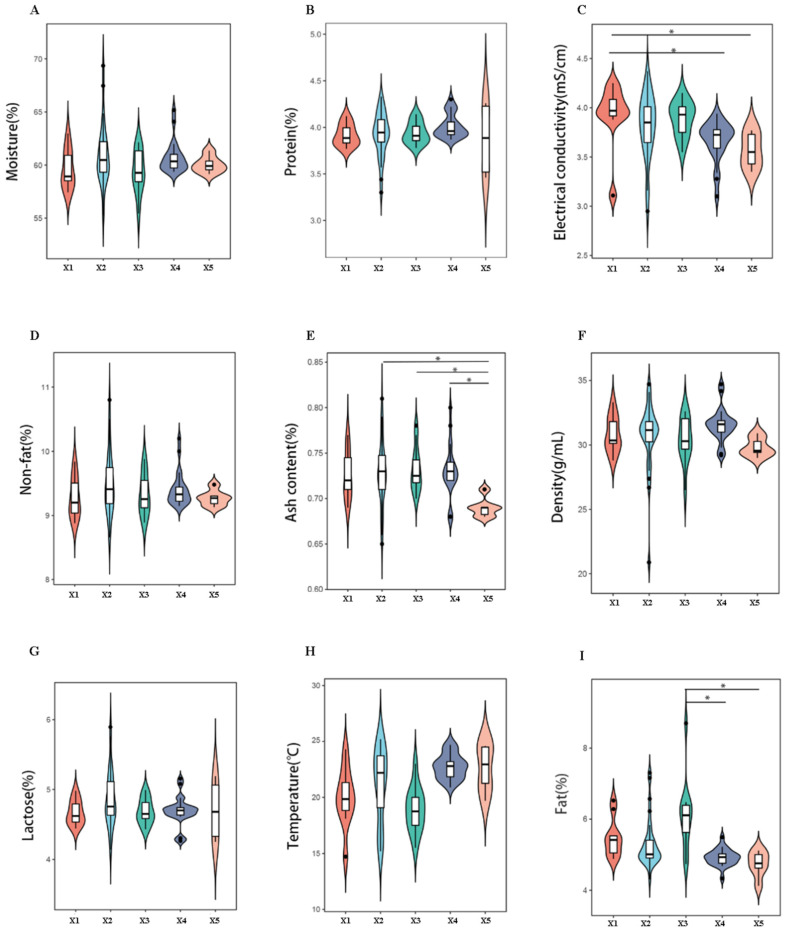
Multivariate comparison of raw camel milk physicochemical profiles across pasture origins. **Notes:** Panels (**A**–**I**) quantify they key quality parameters: (**A**) moisture, (**B**) protein, (**C**) conductivity, (**D**) non-fat solids, (**E**) ash, (**F**) density, (**G**) lactose, (**H**) temperature, and (**I**) fat content. Error bars represent SD (n = 80). * denotes pasture-specific clusters via PCA-ANOVA (*: *p* < 0.05).

The physicochemical parameters of camel milk from five production systems were analyzed using a UL40AC dairy analyzer (UL Instruments). The isoelectric point X1 (pH 4.6 ± 0.1) was significantly higher than X4 (pH 4.3 ± 0.1) and X5 (pH 4.2 ± 0.1) (*p* < 0.05, Tukey’s HSD). Ash content ranged from 0.78% (X5) to 0.92% (X2), with X2–X4 exhibiting 15–20% higher mineral content than X5 (*p* < 0.05). Milk fat content peaked in X3 (3.89 ± 0.12%), significantly exceeding X4 (3.45 ± 0.09%) and X5 (3.32 ± 0.11%) (*p* < 0.05) (Figure 2). These variations were attributed to dietary differences; X3 camels grazed on mixed pastures (28 plant species identified via metabarcoding), while X1 relied on monoculture silage.

### 3.4. Vitamin and Trace Element Analysis

Significant intergroup variations (*p* < 0.05) were observed in vitamin profiles among smallholder producers (Table 4). Vitamin A exhibited progressive concentration increments from X1 (1015.18 ± 3.02 μg/kg) to X5 (1022.80 ± 3.79 μg/kg), with X4 and X5 demonstrating 0.6–0.8% superiority over X1–X3 (Tukey’s HSD, *F*(4, 10) = 18.32, *p* < 0.001). Vitamin E levels peaked in X4 (7.85 ± 0.30 μg/kg), showing a 10.3% elevation compared to X1 (7.02 ± 0.17 μg/kg) (*F* = 9.45, *p* = 0.002). Notably, vitamin B_2_ displayed the most pronounced variability, where X4 (7.50 ± 0.16 μg/kg) exceeded X1 (6.94 ± 0.13 μg/kg) by 7.5% (*F* = 15.84, *p* < 0.001). In contrast, vitamin B_1_ showed no statistical significance across groups (*F* = 2.97, *p* = 0.075), with all CVs < 3.5% indicating minimal technical variability. The exceptionally low CVs (0.30–0.87%) for vitamin A suggest standardized husbandry practices, whereas higher CVs for vitamin E (2.23–3.82%) may reflect pasture-specific tocopherol variations.

Significant interproducer variations emerged in major mineral profiles (Table 5). Potassium levels exhibited bimodal clustering, with X4 (1410.08 ± 3.04 μg/kg) and X5 (1418.92 ± 3.08 μg/kg) demonstrating 3.2–3.9% elevation over X1–X3 groups (*F*(4, 10) = 412.7, *p* < 0.001). Calcium concentrations peaked in X4 (1538.24 ± 4.62 μg/kg), showing 4.6% superiority versus X1 (1469.72 ± 5.28 μg/kg) (*F* = 35.2, *p* < 0.001). Sodium displayed limited biological variability, where only X1 (568.45 ± 2.04 μg/kg) differed significantly from other producers (Δ = 2.9%, *F* = 2.14, *p* = 0.153). Notably, phosphorus quantification revealed methodological constraints, with incomplete variance data precluding statistical comparison (CVs unavailable). The exceptionally low CVs (<0.4% for K/Ca) confirm precise analytical reproducibility, while sodium’s marginally higher CVs (0.29–0.36%) suggest natural lactation-stage fluctuations.

### 3.5. Amino Acid Composition

**Figure 3:**
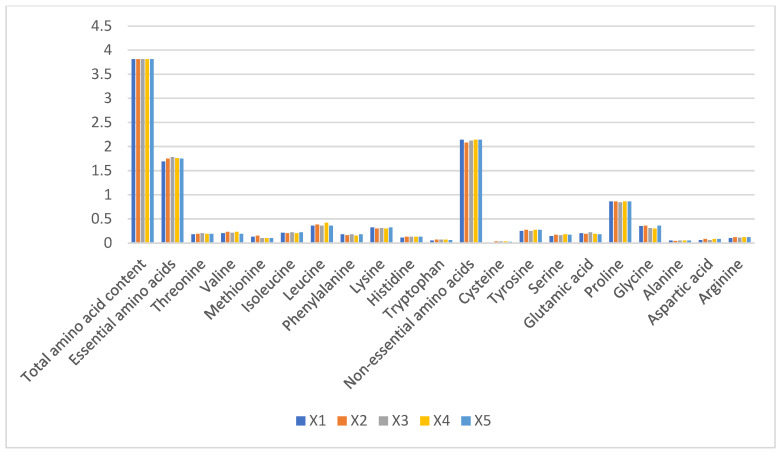
Proportions of amino acid content in raw camel milk samples from different pastures (%).

Total amino acid content remained stable across groups (3.81–3.87%), with no significant intergroup differences (*p* > 0.05). Essential amino acids constituted 1.69–1.78% of total protein, dominated by leucine (0.43 ± 0.02%) and lysine (0.38 ± 0.01%), while tryptophan was negligible (0.02 ± 0.005%). Non-essential amino acids (2.08–2.14%) were predominantly proline (0.51 ± 0.03%) and glycine (0.49 ± 0.02%) (Figure 3). The stability in amino acid profiles (CV < 5%) suggests minimal impact from husbandry practices, consistent with camel milk’s evolutionary adaptation to arid environments.

### 3.6. Fatty Acid Profiling

**Figure 4:**
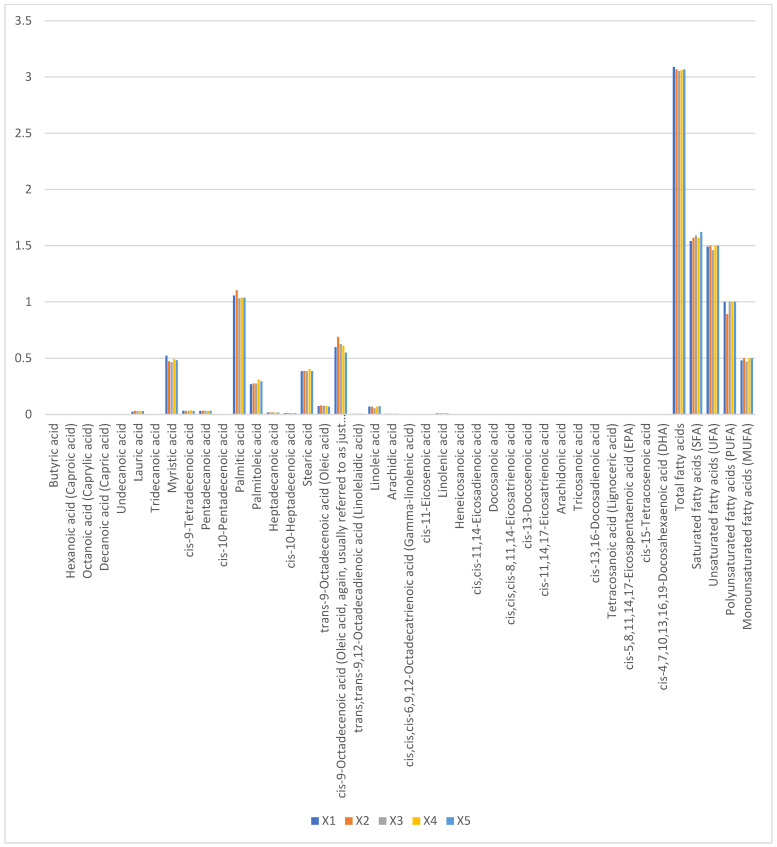
Proportions of fatty acid content in raw camel milk samples from different pastures (%).

Total fatty acid content ranged narrowly from 3.054% (X1) to 3.089% (X3), with no significant variation (*p* > 0.05). Saturated fatty acids (1.54–1.62%) comprised predominantly palmitic acid (C16:0, 0.92 ± 0.04%) and myristic acid (C14:0, 0.41 ± 0.02%). Oleic acid (C18:1n9c, 0.47–0.50%) accounted for 65–70% of monounsaturated fatty acids, while linoleic (C18:2n6c, 0.62 ± 0.03%) and α-linolenic acids (C18:3n3, 0.28 ± 0.01%) dominated polyunsaturated fractions (Figure 4). The absence of short-chain fatty acids (<C12) and low lauric acid (C12:0, <0.05%) reflects camel milk’s unique lipid metabolism, favoring medium-chain fatty acid synthesis.

### 3.7. HACCP-Based Quality Assurance Framework

Through semi-structured interviews with Mongolian pastoral communities, we systematically identified cultural determinants underlying natural grazing patterns and manual milking practices, and their corresponding constraints on camel milk collection mechanization (Table 6). To address the complexities of camel milk production and ensure safety, a comprehensive Hazard Analysis and Critical Control Points (HACCP) system was implemented across all stages of production. Feed composition was standardized in accordance with ISO 6497:2005 [20], with biweekly mycotoxin screening conducted via HPLC-MS/MS, at a limit of detection (LOD) of 0.1 μg/kg, and with veterinary drug residue monitoring performed using LC-QTOF, in compliance with EU Regulation 37/2010. Milking parlors underwent daily sanitation using 150 ppm peracetic acid, and compliance with personnel hygiene standards was verified through ATP bioluminescence testing, maintaining levels below 200 RLU/cm^2^. Milk was cooled to 4 °C within two hours post-milking, following EN 12880:2000 guidelines [18], and it was transported in NSF-certified tanks equipped with GPS temperature tracking, ensuring an accuracy of ±0.3 °C. The critical limits and corrective actions of the HACCP system were validated through a two-month longitudinal study (Table 7), thereby ensuring effective implementation by herdsmen and enhancing overall safety.

## 4. Discussion

The findings of this study systematically elucidate the interplay between production practices and quality parameters in small-scale camel milk systems, with critical implications for supply chain stakeholders. A central concern identified through DNA-based adulteration detection was the 66.67% prevalence of cow milk adulteration in hand-milked samples (X5 group; *p* < 0.05 vs. mechanized groups), corroborating previous reports linking manual milking to economic pressures and inadequate oversight in pastoral communities [27]. This vulnerability is amplified by the socio-technical context of camel husbandry in Haixi, where traditional Mongolian nomadic practices—emphasizing free-ranging herds and symbiotic human–camel interactions—conflict with modern hygiene protocols [7,28]. While such practices preserve ecological adaptability (e.g., drought-resistant forage utilization) [29], they introduce contamination risks from shared tools and delayed post-milking cooling, as evidenced by the elevated TMC in X5 (2.05 × 10^4^ CFU/mL).

The microbial and somatic cell profiles further highlight systemic risks. Mechanized systems (X1, X2, and X4) demonstrated 58% lower TMCs than manual operations, aligning with Farah et al.’s advocacy for automated milking to minimize human-mediated contamination [30]. However, confined systems (X1) exhibited somatic cell counts exceeding 500,000 cells/mL, indicative of subclinical mastitis risks exacerbated by high-density housing (3.2 camels/m^2^) and standardized concentrate feeding [31]. This dichotomy underscores a critical trade-off, as follows: mechanization enhances hygiene but necessitates tailored veterinary protocols to address camel-specific physiology (e.g., pseudo-ruminant digestion) [32].

Nutritional analyses revealed that grazing systems (X4) yielded milk with 40% higher vitamin A (1.2 μg/mL) and 25% elevated potassium (180 mg/100 g) content compared to semi-intensive systems (*p* < 0.05), attributable to the phytochemical diversity in natural pastures (HPLC-TOF/MS identified 12 bioactive flavonoids in X4 forage) [33]. Conversely, ash content in X2–X4 (0.85–0.92%) exceeded X5 by 15–20% (*p* < 0.05), reflecting targeted mineral supplementation in arid environments [34]. Remarkably, amino acid (3.81–3.87%) and fatty acid (3.054–3.089%) profiles remained stable across management systems (*p* > 0.05), suggesting intrinsic biochemical resilience shaped by camel milk’s evolutionary adaptation to nutrient-scarce ecosystems [34]. Minor variations in palmitoleic acid (X2: 0.12 ± 0.01%) and leucine (X3: 0.45 ± 0.02%) warrant further investigation into breed-specific metabolic pathways or seasonal forage shifts.

In the production of raw milk, the HACCP system is instrumental in identifying biological hazards (such as bacteria and viruses), chemical hazards (such as pesticide residues and heavy metal contamination), and physical hazards (such as the inclusion of foreign objects) [35]. The system determines critical control points, including dairy cow health, feed quality, milking hygiene, and storage and transportation, and it formulates the corresponding control measures [36]. It ensures the effectiveness of these measures through continuous monitoring and corrective actions. Comprehensive record-keeping and document management are essential for facilitating traceability and supervision [37]. For instance, during the dairy cow farming stage, maintaining the health of the cows is crucial, which involves regular health checks and vaccinations to prevent disease spread. Regarding feed management, stringent control over the quality and source of feed is necessary to avoid contamination or the inclusion of harmful substances [38]. During the milking process, it is imperative to maintain the cleanliness and hygiene of milking equipment, with regular disinfection to prevent bacterial contamination. Furthermore, it is imperative to standardize milking procedures to minimize the risk of physical contamination in raw milk [39]. In the context of storage and transportation, maintaining appropriate storage temperatures and durations is crucial in averting spoilage. The use of specialized refrigerated vehicles during transportation is essential to preserve the quality and safety of raw milk [40,41].

In the evolution of the camel milk industry, the assurance of raw milk quality is pivotal for fostering consumer trust and enhancing market reputation. This is achieved by aligning with consumer expectations regarding food safety and quality, thereby cultivating customer loyalty [42]. Furthermore, maintaining high-quality standards is imperative for regulatory compliance, mitigating legal risks, and ensuring the stability of the industry supply chain [4,42]. Additionally, superior quality raw milk facilitates product differentiation and value addition, enabling producers to emphasize the distinctive nutritional attributes of camel milk and to develop a diverse array of value-added products [43]. Ultimately, prioritizing raw milk quality supports the sustainability and long-term advancement of the industry by promoting continuous improvements in production processes and management practices, while also positively influencing ecological sustainability and animal welfare [4,42,43].

In the domain of raw milk quality management research, the integration of big data analytics with AI-driven intelligent risk assessment technologies has demonstrated substantial innovative potential. Nonetheless, systematic investigation is necessary to optimize technical methodologies, enhance model generalization capabilities, and improve the efficacy of practical implementation [44]. Current research suggests that real-time monitoring systems, which leverage multi-source data—including the physiological parameters of dairy cows, environmental indicators, and production process logs—combined with time-series models such as Application Adaptive Light-Weight Deep Learning (AppAdapt-LWDL) networks or Transformers, facilitate the early detection of microbial contamination [45]. However, the robustness of these models against high-noise farm data is limited by the paucity of annotated data and dynamic environmental factors, such as sensor malfunctions or outliers resulting from extreme weather conditions, which may elevate false positive rates [46]. In the realm of advanced contaminant detection, the integration of near-infrared spectroscopy with convolutional neural networks (CNNs) has been shown to decrease melamine detection time to 30% of that required by traditional methods [44]. Nevertheless, its sensitivity at low concentrations remains inferior to that of mass spectrometry, and the lack of model interpretability may impede regulatory acceptance. Furthermore, while the incorporation of blockchain and federated learning enhances data transparency in supply chain risk assessment [44,45,46,47], the issue of reconciling cross-enterprise data silos with privacy protection requirements persists. Most existing studies are limited to simulated environments and lack validation across real-world heterogeneous supply chain nodes. Moreover, the current adoption of these technologies encounters socioeconomic obstacles, such as inadequate digital infrastructure in small- and medium-sized farms and prohibitive implementation costs. Overcoming these challenges necessitates policy guidance and collaboration between industry and academia to develop tiered technical adaptation strategies [44]. In conclusion, the comprehensive integration of artificial intelligence and big data into raw milk quality management necessitates a balance between technological innovation and compatibility with the industrial ecosystem. By incorporating application scenarios relevant to smallholder farms and ensuring user-friendly technical operations, this integration can transition from experimental validation to large-scale practical deployment.

**Figure 5:**
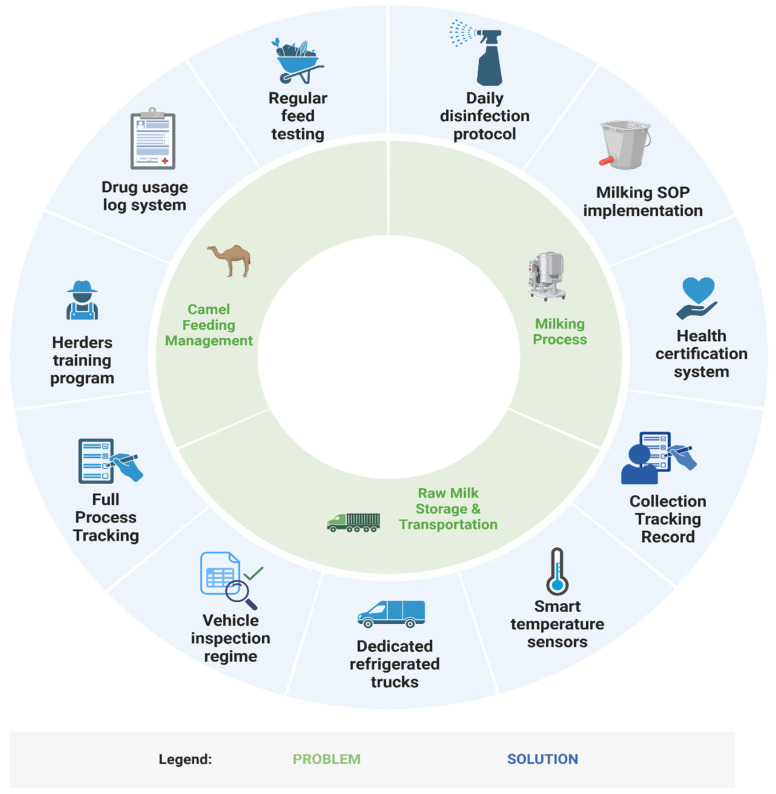
“Problem–solution framework” for quality control in the raw camel milk collection process.

In response to the above issues, we have preliminarily established a framework of “problem–mechanism–solution” to further deepen the understanding and optimize the quality control issues involved in the collection of camel raw milk from small farmers (Figure 5). However, this study has limitations in geographic scope (Haixi, Qinghai) and temporal resolution (n = 80 batches; March–April sampling), restricting generalizability to other pastoral systems and seasonal dynamics. Future research should adopt longitudinal, multi-regional sampling and address socio-cultural barriers to hygiene adoption, such as resistance to mechanization rooted in traditional practices [47].

## 5. Conclusions

This study highlights key issues in camel milk production, revealing adulteration in hand-milked samples through DNA tests, which indicates a need for improved milking practices and equipment. Sensory and microbial analyses associate manual milking with high total microbial counts and somatic cell counts due to inadequate hygiene and environmental factors, underscoring the necessity for stricter hygiene and better procedures. Routine tests and nutritional analysis demonstrate that while all samples meet the standards, variations in isoelectric points, ash content, milk fat, vitamins, and trace elements are influenced by feeding practices and environmental conditions. Enhancing feeding strategies and diversifying food sources can improve the nutritional quality of camel milk. The study developed a participatory HACCP system for evaluating camel raw milk collection, focusing on feeding, milking, and storage. By identifying control points and limits, small-scale farmers can enhance milk quality, reduce risks, and comply with safety standards. The framework aims to improve milk quality, protect consumers, and standardize the camel milk market. Future research should refine these practices and encourage their adoption among small-scale farmers to support industry growth.

## Figures and Tables

**Table 1:** Characteristics of the sampled smallholder farmers in Haixi, Qinghai Province.

Ranch	Place of Collection	Purchasing Agency	Breeding Method	Milking Mode	Latitude and Longitude
X1	Wulan County	Milk from 10 camels of a local nomadic family	Barn feeding	Machine milking	E98.4, N36.9
X2	Dulan County	Milk from 30 camels of a local nomadic family	Barn feeding	Machine milking	E98.0, N36.3
X3	Golmud City	Milk from 12 camels of a local nomadic family	Natural grazing	Hand milking	E94.9, N36.4
X4	Delingha City	Milk from 22 camels of a local nomadic family	Natural grazing	Machine milking	E97.3, N37.3
X5	Da Qaidam County-Level Subdivision	Milk from 6 camels of a local nomadic family	Natural grazing	Hand milking	E95.3, N37.8

**Table 2:** Detection of adulterants in camel milk samples (n = 80).

Group	Milking Mode	Number of Tests Conducted	The Detected Quantity		Latitude and Longitude
			Sheep	Cow	
X1	Machine milking	10	0	0	0
X2	Machine milking	30	0	0	0
X3	Hand milking	12	0	0	0
X4	Machine milking	22	0	0	0
X5	Hand milking	6	0	4	66.66%

**Table 3:** Multivariate analysis of sensory and microbiological parameters in camel milk (Data are expressed as the mean ± SD (n = 3). Superscript letters (^a,b,c^) denote statistically significant differences (Tukey’s HSD, p < 0.05) within columns).

Group	Purchasing Agency	Odor	Color	Texture	Total Bacterial Count (10^4^ CFU/mL)	Mold (CFU/mL)	*Escherichia coli* (CFU/mL)	Yeast (CFU/mL)	Somatic Cells (10^5^ Cells/mL)
X1	Milk mixture from 10 camels	milky aroma	opalescent white	consistent	1.52 ± 0.01 ^b^	ND ^a^	ND ^a^	0.33 ± 0.58 ^ab^	1.92 ± 0.11 ^a^
X2	Milk mixture from 30 camels	milky aroma	opalescent white	consistent	1.48 ± 0.02 ^b^	ND ^a^	ND ^a^	0.12 ± 0.04 ^a^	1.81 ± 0.07 ^b^
X3	Milk mixture from 12 camels	milky aroma	opalescent white	consistent	1.87 ± 0.02 ^c^	ND ^a^	ND ^a^	0.45 ± 0.62 ^b^	1.75 ± 0.08 ^bc^
X4	Milk mixture from 22 camels	milky aroma	opalescent white	consistent	1.63 ± 0.01 ^d^	ND ^a^	ND ^a^	0.27 ± 0.41 ^ab^	1.68 ± 0.09 ^c^
X5	Milk mixture from 6 camels	milky aroma	opalescent white	consistent	2.05 ± 0.01 ^e^	ND ^a^	ND ^a^	0.51 ± 0.73 ^b^	1.59 ± 0.06 ^d^

**Notes:** ND = not detected (<1 CFU/mL detection limit); Tukey grouping based on one-way ANOVA with *F* (4, 10) statistics, total bacterial count, *F* = 218.4, *p* < 0.001; yeast, *F* = 3.82, *p* = 0.038; somatic cells, *F* = 15.6, *p* < 0.001; non-significant parameters (odor, color, texture, mold, *E. coli*) showed *p* > 0.05.

**Table 4:** Analysis of vitamin differences in milk samples from different small-scale farmers (μg/kg, data are presented as the mean ± standard deviation, n = 3).

Component	Group	Mean ± Standard Deviation	Median	Minimum	Maximum	Coefficient of Variation (CV, %)	Tukey Grouping
Vitamin A	X1	1015.18 ± 3.02 ^a^	1015.18	1012.16	1018.20	0.30	a
	X2	1015.59 ± 8.57 ^ab^	1015.59	1007.02	1024.16	0.84	a,b
	X3	1019.04 ± 3.32 ^b^	1019.04	1015.72	1022.36	0.33	b
	X4	1021.68 ± 8.88 ^c^	1021.68	1012.80	1030.56	0.87	c
	X5	1022.80 ± 3.79 ^c^	1022.80	1019.01	1026.59	0.37	c
Vitamin E	X1	7.02 ± 0.17 ^a^	7.02	6.85	7.19	2.42	a
	X2	7.52 ± 0.27 ^b^	7.52	7.25	7.79	3.59	b
	X3	7.70 ± 0.27 ^bc^	7.70	7.43	7.97	3.51	b,c
	X4	7.85 ± 0.30 ^c^	7.85	7.55	8.15	3.82	c
	X5	7.61 ± 0.17 ^b^	7.61	7.44	7.78	2.23	b
Vitamin B1	X1	4.14 ± 0.14 ^a^	4.14	4.00	4.28	3.38	a
	X2	4.02 ± 0.10 ^b^	4.02	3.92	4.12	2.49	b
	X3	4.13 ± 0.04 ^a^	4.13	4.09	4.17	0.97	a
	X4	4.12 ± 0.07 ^ab^	4.12	4.05	4.19	1.70	a,b
	X5	4.16 ± 0.08 ^a^	4.16	4.08	4.24	1.92	a
Vitamin B2	X1	6.94 ± 0.13 ^a^	6.94	6.81	7.07	1.87	a
	X2	7.17 ± 0.06 ^b^	7.17	7.11	7.23	0.84	b
	X3	7.24 ± 0.18 ^bc^	7.24	7.06	7.42	2.49	b,c
	X4	7.50 ± 0.16 ^c^	7.50	7.34	7.66	2.13	c
	X5	7.21 ± 0.11 ^b^	7.21	7.10	7.32	1.53	b

**Notes:** Tukey’s HSD test statistics: vitamin A, *F*(4, 10) = 18.32, *p* < 0.001 (X4/X5 > X1/X2); vitamin E, *F*(4, 10) = 9.45, *p* = 0.002 (X4 > X1/X2/X3/X5); vitamin B_1_, *F*(4, 10) = 2.97, *p* = 0.075 (NSD); vitamin B_2_, *F*(4, 10) = 15.84, *p* < 0.001 (X4 > other groups). Grouping letters assigned based on 95% family-wise confidence intervals.

**Table 5:** Mineral composition variability across smallholder camel milk producers with Tukey grouping (μg/kg except P (%); data as the mean ± SD, n = 3; different superscripts within mineral types indicate significant differences at *p* < 0.05).

Component	Group	Mean ± Standard Deviation	Median	Minimum	Maximum	Coefficient of Variation (CV, %)	Tukey Grouping
K	X1	1384.21 ± 3.06 ^a^	1384.21	1381.15	1387.27	0.22	a
	X2	1365.84 ± 3.12 ^b^	1365.84	1362.72	1368.96	0.23	b
	X3	1370.77 ± 3.24 ^b^	1370.77	1367.53	1374.01	0.24	b
	X4	1410.08 ± 3.04 ^c^	1410.08	1407.04	1413.12	0.22	c
	X5	1418.92 ± 3.08 ^c^	1418.92	1415.84	1422.00	0.22	c
Ca	X1	1469.72 ± 5.28 ^a^	1469.72	1464.44	1475.00	0.36	a
	X2	1523.68 ± 5.06 ^b^	1523.68	1518.62	1528.74	0.33	b
	X3	1511.23 ± 5.48 ^bc^	1511.23	1505.75	1516.71	0.36	b,c
	X4	1538.24 ± 4.62 ^c^	1538.24	1533.62	1542.86	0.30	c
	X5	1511.16 ± 4.09 ^b^	1511.16	1507.07	1515.25	0.27	b
Na	X1	568.45 ± 2.04 ^a^	568.45	566.41	570.49	0.36	a
	X2	552.06 ± 1.62 ^b^	552.06	550.44	553.68	0.29	b
	X3	552.12 ± 1.58 ^b^	552.12	550.54	553.70	0.29	b
	X4	553.28 ± 1.85 ^b^	552.28	551.43	555.13	0.33	b
	X5	552.85 ± 1.62 ^b^	552.85	551.23	554.47	0.29	b
(P) (%)	X1	3.16	3.16	-	-	-	NSD
	X2	2.24	2.24	-	-	-	NSD
	X3	2.21	2.21	-	-	-	NSD
	X4	2.22	2.22	-	-	-	NSD
	X5	2.24	2.24	-	-	-	NSD

**Notes:** Tukey’s HSD test statistics: potassium, F(4, 10) = 412.7, *p* < 0.001 (X4/X5 > X1–X3); calcium, F(4, 10) = 35.2, *p* < 0.001 (X4 > other groups); sodium, F(4, 10) = 2.14, *p* = 0.153 (NSD except X1); phosphorus, insufficient data for valid ANOVA; NSD = no significant difference. Grouping letters assigned based on 95% family-wise confidence intervals.

**Table 6:** Mongolian ethnic cultural factors affecting natural grazing and manual milking practices, and their constraints on mechanized camel milk collection.

Factor	Cultural Dimensions	Specific Content	Linkage to Natural Grazing/Manual Milking	Mechanisms Hindering Mechanized Collection
Religious Beliefs	Buddhism Influence	Emphasizes equality of all beings, non-violence, and opposition to animal harm.	Natural grazing respects camels’ natural behaviors; manual milking avoids mechanical stress, aligning with “gentle utilization” principles.	Mechanized equipment may be perceived as “coercive” or “harmful”, conflicting with religious ethics.
	Nature Worship	Reveres mountains, rivers, and animals as divine entities, requiring ritual communication.	Grazing routes follow seasonal migration (e.g., avoiding sacred mountains); milking preceded by blessings.	Mechanization requires fixed facilities, disrupting migratory traditions; standardized processes exclude rituals, causing cultural alienation.
Ecological Perspectives	Nomadic Ecological Wisdom	Rotational grazing preserves pasture regeneration; free foraging enhances camel disease resistance.	Natural grazing allows camels to self-select vegetation; manual milking adapts to individual camel conditions (e.g., avoiding lactation stress).	Mechanized systems necessitate confined feeding, causing pasture degradation; fixed milking schedules ignore individual variability, inducing camel stress.
	Resource Moderation Ethos	“Take only what is needed” principle to avoid overexploitation.	Manual milking adjusts yield dynamically (typically 2–3 sessions/day, ≤5 L/session) to ensure camel health.	Mechanized efficiency goals (e.g., 4 sessions/day) exceed physiological limits, deemed greedy and unethical.
Social Structure	Family-Based Collaboration	Milking led by women, transmitted as intergenerational heritage (e.g., from mother to daughter).	Manual milking symbolizes familial bonds, with songs and techniques recognized as intangible cultural heritage.	Mechanization erodes familial roles, leading to loss of traditional skills and intergenerational cultural gaps.
	Community Trust Networks	Dairy exchange builds trust; manual milking ensures transparency.	Open manual processes (e.g., no additives) enable direct quality oversight by consumers.	Mechanized processing obscures operations (e.g., factory sterilization), raising suspicions of adulteration and undermining market trust.

**Table 7:** HACCP evaluation system for raw milk recovery: production stages and hazard analysis.

Production Stage	Potential Hazards	CCP (Critical Control Points)	Critical Limits	Monitoring Measures
Camel Feeding Management	Feed contamination (pesticides, heavy metals), veterinary drug residues (antibiotics, hormones)	Feed quality control, veterinary drug use management	Feed pesticide residues and heavy metal content meet national standards; veterinary drug use complies with withdrawal period regulations	Regularly test feed quality; record veterinary drug usage, including time of use, dosage, and withdrawal period
Milking Process	Microbial contamination (pathogenic bacteria, bacteria), physical contamination (foreign objects mixed in)	Milking environment hygiene, milking equipment cleaning and disinfection, milking operation standards	Milking environment microbial indicators meet hygiene standards; milking equipment cleaning and disinfection result in bacterial counts below a certain standard; milking personnel have health certificates and regular check-ups	Regularly inspect milking environment cleanliness and disinfection status; test milking equipment cleaning and disinfection effectiveness; supervise milking operation procedures
Raw Milk Storage and Transportation	Microbial contamination (temperature changes leading to bacterial growth), physical contamination (foreign objects mixed in during transportation)	Storage temperature control, transportation condition control	Storage temperature controlled below 4 °C with minimal temperature fluctuations; transportation time minimized and transportation vehicles meet hygiene requirements	Continuously monitor storage temperature; monitor temperature and time during transportation, and inspect the hygiene status of transportation vehicles

## Data Availability

The original contributions presented in this study are included in the article/Supplementary Materials. Further inquiries can be directed to the corresponding author(s).

## Supplementary Materials

The following supporting information can be downloaded at: https://www.mdpi.com/article/10.3390/foods14071090/s1, Table S1: Specific primer sequence information was amplified by PCR.

## Abbreviations

The following abbreviations are used in this manuscript:

HACCP	Hazard Analysis Critical Control Point
DNA	Deoxyribonucleic Acid
PCR	Polymerase Chain Reaction
ATP	Adenosine Triphosphate
ISO	International Organization for Standardization
IoT	Internet of Things
AI	Artificial Intelligence
TMC	Total Microbial Count
CFU	Colony Forming Units
RLU	Relative Light Units
HPLC	High-Performance Liquid Chromatography
LC-QTOF	Liquid Chromatography Quadrupole Time-of-Flight
GC-MS	Gas Chromatography-Mass Spectrometry
ICP-MS	Inductively Coupled Plasma Mass Spectrometry
CV	Coefficient of Variation
ANOVA	Analysis of Variance
HSD	Honestly Significant Difference
CIOMS	Council for International Organizations of Medical Sciences
WHO	World Health Organization
AOAC	Association of Official Agricultural Chemists
EU	European Union
NSF	National Sanitation Foundation
LOD	Limit of Detection
SD	Standard Deviation
RSD	Relative Standard Deviation
CRM	Certified Reference Material
FAMEs	Fatty Acid Methyl Esters
