# Supplementary material for: Risk Prevention and Quality Control in Camel Milk Collection: Insights from Field Research
Source: Foods. 2025 Mar 21;14(7):1090. doi: 10.3390/foods14071090 (PMC11988837; doi:10.3390/foods14071090)
Supplement: Supplementary file 1 [file foods-14-01090-s001.zip › foods-3508004-supplementary.pdf]

Supplementary Table S1. Specific primer sequence information was amplified by PCR.

Primer	Primer sequence (5' to 3')	Gene	Length (bp)
Camel-specific primers	F: CATTATCACGGCTCTAGTGGC<br>R:CTGGTGAGAATAATACGAGGATAAG	Cyt b	182
Sheep-specific primers	F: GAGTAATCCTCCTATTTGCGAC<br>R: GAACTATGGCGAGGGCTGC	Cyt b	240
Bovine-specific primers	F: GTACTATTTGCGCCCAACCTCC<br>R: AGAACAGGCATTGGCTGAGCA	Cyt b	250
